# Proteomic analysis during of spore germination of *Moniliophthora perniciosa*, the causal agent of witches’ broom disease in cacao

**DOI:** 10.1186/s12866-017-1085-4

**Published:** 2017-08-17

**Authors:** Joise Hander Mares, Karina Peres Gramacho, Everton Cruz Santos, André da Silva Santiago, Juliano Oliveira Santana, Aurizângela Oliveira de Sousa, Fátima Cerqueira Alvim, Carlos Priminho Pirovani

**Affiliations:** 1 Laboratory of Proteomics, Center of Biotechnology and Genetics, State University of Santa Cruz (UESC), Ilhéus, Bahia Brazil; 2 Laboratory of Plant Pathology, Cacao Research Center, CEPLAC, Ilhéus, Bahia Brazil; 3 Center for Molecular Biology and Genetic Engineering (CBMEG) Unicamp, São Paulo, Brazil

**Keywords:** Basidiospore, Germination, Witches’ broom, Proteomic analysis

## Abstract

**Background:**

*Moniliophthora perniciosa* is a phytopathogenic fungus responsible for witches’ broom disease of cacao trees (*Theobroma cacao* L.). Understanding the molecular events during germination of the pathogen may enable the development of strategies for disease control in these economically important plants. In this study, we determined a comparative proteomic profile of *M. perniciosa* basidiospores during germination by two-dimensional SDS-PAGE and mass spectrometry.

**Results:**

A total of 316 proteins were identified. Molecular changes during the development of the germinative tube were identified by a hierarchical clustering analysis based on the differential accumulation of proteins. Proteins associated with fungal filamentation, such as septin and kinesin, were detected only 4 h after germination (hag). A transcription factor related to biosynthesis of the secondary metabolite fumagillin, which can form hybrids with polyketides, was induced 2 hag, and polyketide synthase was observed 4 hag. The accumulation of ATP synthase, binding immunoglobulin protein (BiP), and catalase was validated by western blotting.

**Conclusions:**

In this study, we showed variations in protein expression during the early germination stages of fungus *M. perniciosa*. Proteins associated with fungal filamentation, and consequently with virulence, were detected in basidiospores 4 hag., for example, septin and kinesin. We discuss these results and propose a model of the germination of fungus *M. perniciosa*. This research can help elucidate the mechanisms underlying basic processes of host invasion and to develop strategies for control of the disease.

**Electronic supplementary material:**

The online version of this article (doi:10.1186/s12866-017-1085-4) contains supplementary material, which is available to authorized users.

## Background

*Moniliophthora perniciosa* is a phytopathogenic fungus that belongs to class Basidiomycetes, order Agaricales, family Marasmiaceae [1]. This fungus causes witches’ broom disease of cacao trees (*Theobroma cacao L.*) and caused the loss of ~90% of cacao harvest in Central and South America in 1990 [2]. *M. perniciosa* is classified as hemibiotrophic, having two stages in its life cycle, the biotrophic (parasitic) phase with monokaryotic hyphae, and the necrotrophic phase (saprophytic) with dikaryotic hyphae containing clamp connections [3]. Basidiospores are the infective propagules and can penetrate the host directly through the intact cuticle, natural openings of the cuticular surface, via epidermal cell junctions, at the base of trichomes, or through stomata [4, 5]. The mycelium forms as hyphae grow within the plant, penetrating the plant tissue in direct contact with the host protoplasm; alternatively, mycelium can form on the plant surface [6]. Recent observations suggest that penetration by the basidiospore is related to the formation of a germination pore directly in the meristematic cuticle, followed by the emergence of a germinative tube [5]. During this infection phase, the fungus induces drastic physiological and morphological changes in the plant [7, 8]. After 2 to 3 months, the infected tissue enters a state of necrosis, and the affected branch of the plant dries and resembles a broom; this is the characteristic feature after which the disease is named [3, 9].

Accession and germination of fungal spores on the surface of plants are the initial steps essential for host penetration and colonization. Some studies have focused on molecular analysis of the germination of conidia and other types of phytopathogenic-fungi spores. Differentially expressed genes were identified in teliospores of *Ustilago maydis*, [10], in conidia of *Fusarium graminearum* [11], and in *Botrytis cinerea* [12]. In addition, analysis of protein profiles of filamentous-fungi spores identified proteins involved in the metabolism of carbohydrates, lipids, and proteins in *Blumeria erysiphe* [13]. Analysis of early stages of germination shows high expression of proteins associated with energy metabolism in conidia of both *Aspergillus nidulans* [14] and *Colletotrichum acutatum* [15]. Understanding the molecular events during germination may enable better strategies for disease control in these economically important plants. Nonetheless, few studies have been conducted to characterize and identify proteins involved in the germination of phytopathogenic fungi such as *M. perniciosa*.

The function of antioxidant proteins in spore survival has been barely reported. Nevertheless, some reports have revealed that enzymes involved in the stress response, e.g., superoxide dismutase (SOD) and catalase, show altered expression in *Aspergillus niger* conidiospores under temperature stress [16].

Modifications of expression patterns of heat shock proteins like binding immunoglobulin protein (BiP) may modulate the virulence of *Pyrenophora tritici-repentis*, the causal agent of tan spot of wheat [17]. A comparative analysis of proteins secreted by *Verticillium dahlia* during host invasion indicates that ATP levels change during infection [18]. Additionally, BiP seems to be involved in the virulence of other pathogens such as *Bartonella henselae* during host invasion [19].

Recently, our team developed methods for proteomic studies of *M. perniciosa* spores [20]. A protein extraction method and the quantity of basidiocarps and basidiospores required for proteomic studies were established, which allowed for the construction of a reference map based on two-dimensional (2D) polyacrylamide gel electrophoresis (PAGE). Next, a network of protein interactions was built through identification of proteins in nongerminated spores. In the present study, we determined the comparative proteomic profile of germinated and nongerminated basidiospores. In addition to the 175 protein spots identified by coincidence with the reference map for nongerminated-spore proteins [20], we identified >141 unique protein spots seen 2 and 4 h after germination (hag). Molecular changes during the development of the germinative tube were identified by a hierarchical clustering analysis based on differential accumulation of proteins.

## Methods

### Cultivation and collection of basidiospores

Approximately 300 dry branches of a susceptible cacao cultivar (Catongo) between 30 and 50 cm long that were infected with the rosy mycelium of *M. perniciosa* [20] were randomly collected in the field (CEPLAC/CEPEC-Ilhéus, Bahia) according to the methodology described by Mares and coworkers [20]. Basidiospores were produced by the method of Frias et al. [21] and stored in liquid nitrogen as described by Frias [22] until use.

### Germination of basidiospores and protein extraction

Each aliquot of basidiospores was defrosted on ice, diluted in distilled water to reduce the concentration of glycerol to 3%, and incubated in the dark at 25 °C. Germination rates and lengths of the germinative tubes were monitored and measured with a graduated lens under a light microscope (Bioval L2000A) at 40× magnification.

The 0-, 2-, and 4-hag. samples were collected by centrifugation for 20 min at 5000 × *g* in 3% glycerol. The supernatants were discarded, then the precipitates were snap-frozen in liquid nitrogen and stored at −80°C until protein extraction. The samples stored at −80°C were defrosted and washed with cold acetone containing 0.07% v⋅v^−1^ β-mercaptoethanol. Then, protein extraction and quantification were performed according to methods described by Mares and coworkers [20].

### 2D electrophoresis

For proteomic analysis by 2D PAGE, 350 μg of total protein diluted in 250 μL of the rehydration solution (7 mol.L^−1^ urea, 2 mol.L^−1^ thiourea, 2% CHAPS, and 0,002% bromophenol blue) was used. Gel strips (containing immobiline) 13 cm long with an immobilized nonlinear pH gradient from 3 to 10 (Amersham Biosciences, Immobiline™ Dry-Strip) were hydrated for 12 h in the rehydration buffer containing the total protein solution by the methods of Mares and coworkers [20] in an Ettan IPGphor 3 Isoelectric Focusing System. The second dimension was resolved in a 12.5% polyacrylamide gel in a HOEFER SE 600 Ruby vertical electrophoresis system (Amersham Bioscience). The run started with an electrical current of 15 mA per gel for 15 min, followed by 30 μA per gel for 30 min, and 50 μA per gel for 3.5 h, totaling 4.25 h. The gels were prepared in triplicate for each time stage hours after the start of germination.

#### Visualization of spots and image analysis

After electrophoresis, the polyacrylamide gels were placed in fixation buffer (40% ethanol and 10% acetic acid) for 1 h, then the buffer was replaced with colloidal Coomassie Blue dye (8% ammonium sulfate, 0.8% phosphoric acid, 0.08% Coomassie Brilliant Blue G-250, and 20% methanol), and the gels were incubated for 5 days with gentle rocking. The dye was replaced with distilled water, and the gentle rocking of the gels was continued with daily water changes until the excess dye was removed.

Gel images were scanned with a Labscanner (Amersham Bioscience) and analyzed for detection and relative quantification of protein spots using the ImageMaster 2D Platinum 7.0 software (GE Healthcare), considering the area and intensity of the spots. The reference gels (master gel) from triplicates were used to identify the unique spots and compare the relative accumulation of proteins present in the different treatment groups. The spots previously identified in the reference map [20] were compared with the spots on gels for 2 and 4 hag. As for the spots shared among different treatment groups, those that showed a relative fold change in abundance greater than 1.5 were designated as differentially expressed, and statistical significance was determined if *p* < 0.05 in ANOVA. The gels from 2- and 4-h treatment groups were compared with the gels of nongerminated spores obtained by Mares and colleagues [20], which served as a reference (Additional files 1 and 2: Supplementaty Tables 1 and 2).

### Protein identification by mass spectrometry

The spots differentially expressed between 2 and 4 hag. were excised from gels and placed in microtubes. Then, they were bleached and subjected to trypsin digestion [23]. Next, the samples containing recovered peptides were vacuum concentrated until they reached a volume of 10–15 μL.

The resulting peptides from tryptic digestion were subjected to liquid chromatography with tandem mass spectrometry (LC-MS/MS) on a nanoAcquity system (Waters, Milford, MA) coupled with a Q-ToF micro mass spectrometer (Waters), according to methods described elsewhere [23]. The raw data were processed, and the resulting spectra were analyzed in the ProteinLynx Global Server 4.2 software (Waters) and compared with the SwissProt database (http://www.uniprot.org/downloads, October 2011). For comparison with the NCBI database, the MASCOT tool MS/MS IonSearch (www.matrixscience.com) was used with the following settings: tryptic digestion, with 1 cleavage site lost, cysteines modified by carbamidomethylation and methionine oxidation, error tolerance for the peptide of 30 ppm, and fragment mass error of MS/MS equal to 0.1 Da. According to MASCOT analysis probability, only the significant “hits” (*p* < 0.05) were accepted. After protein identification, their ontology and biological processes were classified in Blast2Go.

### Validation of proteomic analysis by western blotting

Aliquots of 10 μg of total basidiospore protein (0, 2, and 4 hag) were separated by 12.5% SDS-PAGE and transferred to a nitrocellulose membrane using the iBlot Dry Blotting System (Invitrogen). The membrane was blocked with 5% skim milk (*w*/*v*) in TBS-T buffer (100 mmol⋅L^−1^ Tris-HCl, pH 8.0; 140 mmol⋅L^−1^ NaCl; 0.05% v⋅v^−1^ Tween 20). BiP (71 kDa), catalase (83 kDa), and ATP synthase (53 kDa) were detected using polyclonal primary antibodies (at 1:2000 dilution) against the following proteins: BiP of *Arabidopsis thaliana* (Agrisera-AS09481), catalase of *A. thaliana* (501,100 Agrisera-AS09), and ATP synthase of *A. thaliana* (Agrisera-AS05085). The membranes were incubated with the appropriate primary antibody for 60 min. After three washes with TBS-T buffer, the membranes were incubated for 60 min with a secondary antibody: a goat anti-rabbit IgG antibody conjugated with alkaline phosphatase (Thermo Fisher Scientific-65-6122). The phosphate of 5-bromo-4-chloro-3-indolyl (BCIP) and p-nitrotetrazolium (NBT; Promega, USA) served as substrates for the colorimetric reaction of alkaline phosphatase. The accumulation of proteins BiP, catalase, and ATP synthase was quantified by means of the membrane images using the GelQuant.Net 1.8.0 software (www.biochemlabsolutions.com). To confirm equal distribution of proteins, the membranes were washed with 0.1 M glycine, pH 2.9, for western blotting membrane stripping, and a new labeling was made. The revelation time was increased until background bands shows up to indicates the homogeneous transfer among the samples.

### Hierarchical clustering analysis

Clustering was performed using the Cluster 3.0 + Java TreeView software (http://bonsai.hgc.jp/~mdehoon/software/cluster/software.htm). For this purpose, the matrix was built from normalized log-transformed ratios for each protein spot from the analysis of gel images in the Image Master 2D Platinum 7.0 software (GE Healthcare); (Additional file 1: Table S1). Euclidean distance (ED) was used to calculate the distance or dissimilarity between individuals and Complete Link was used for clustering.

## Results and discussion

### The protein profile according to 2D SDS-PAGE

Previously, we established a reference map for proteins in nongerminated *M. perniciosa* spores [20]. In the present study, we determined the protein profiles of *M. perniciosa* spores 2 and 4 hag. and compared them with the previously generated data. These collect points were demonstrated as being the necessary time to complete translocation of the intracellular content from the spore to germinative pore. This was noted due to the lack of staining of the basidiospore 4 after the germination, which is considered complete germination [20].

A total of 141 proteins were identified by mass spectrometry, totaling 319 when combined with those identified by Mares and colleagues [20]. The number of proteins identified in other proteomic studies included 118 in *Botrytis cinerea* spores [24], 130 in *Rhizoctonia solani* AG-1 sclerotia maturation [25, 26], and 365 in *Colletotrichum acutatum* conidia germination [15]. Thus, the present study identified one of the largest numbers of proteins in spores of a phytopathogenic fungus. In addition, the accumulation of proteins BiP, catalase A, and ATP synthase β subunit was validated by western blotting with antibodies against the *A. thaliana* proteins. The homology of these *M. perniciosa* proteins with the corresponding *A. thaliana* proteins is 66, 48, and 66%, respectively. Alignments between the proteins of these two species showed blocks of conserved amino acid residues long enough for the presence of reactive epitopes that can be detected by polyclonal antibodies (Additional file 2: Figure S1). Next, we will discuss the comparative proteomic profiles corresponding to the germination of *M. perniciosa* basidiospores.

**Fig. 1:**
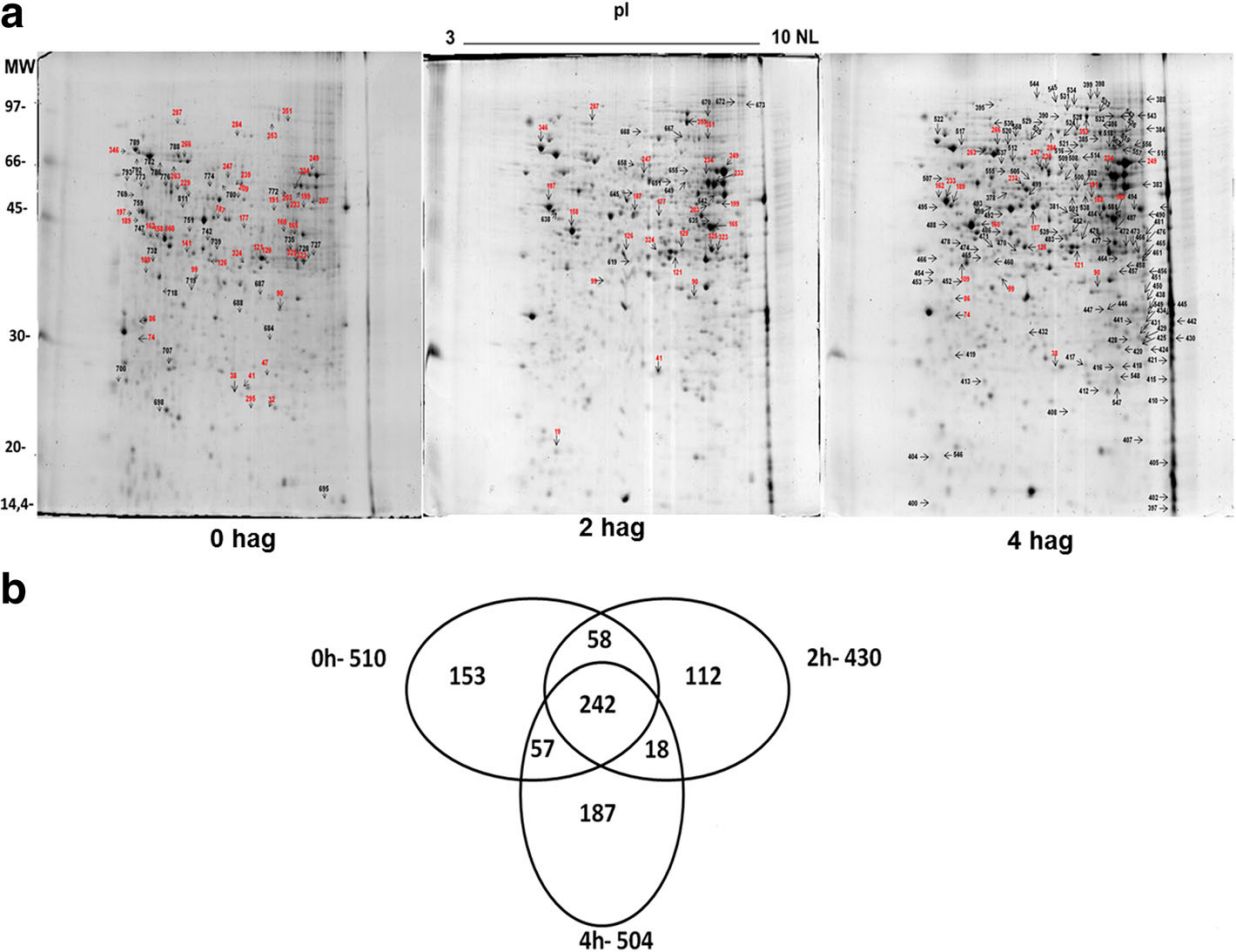
Protein profile in 2DE-PAGE at different stages of *M. perniciosa* spore germination. **a** Non-germinated basidiospores of *M. perniciosa* (0 h) (Mares, et al., [20]) with 2 and 4 h after germination. The samples were focused in strips of 13 cm with nonlinear (NL) pH gradient 3–10. Black numbers correspond to unique spots for each treatment. Red numbers correspond to spots common between treatments with fold change >1.5. **b** Venn diagram of the distribution of spots detected in gels of 0 (Mares et al., [20]), 2, and 4 h after basidiospore germination

2D gels of basidiospore proteins for germination time points 0, 2, and 4 h were analyzed for variation in abundance and distribution of spots to obtain protein profiles (Fig. 1a). Proteins were visualized in the whole pH range, and molecular weight (MW) was noted. A greater abundance of proteins with MW above 90 kDa is seen in the 2- and 4-hag. samples as compared to baseline (Fig. 1a). The gel analysis revealed 510, 430, and 504 spots for the germination time points 0, 2, and 4 h, respectively. A total of 242 spots were common among all the three treatment groups (Fig. 1b). Thirty percent (153 spots) of spots identified in nongerminated basidiospores were unique to this group. Basidiospores at 2 hag. had 26% (112) unique spots. Basidiospores at 4 hag. had 37% (187) unique spots.

All the treatment groups were evaluated concerning the distribution of spots relative to their MW and isoelectric point (pI; Additional file 3: Figure S2 and Additional file 4: Figure S3). Most proteins that constitute the proteomic map for the three time points under study are distributed in the range of 30 to 60 kDa (63.4, 64.8, and 53.5% for 0, 2, and 4 h, respectively). A 31.5% increase was observed in the percentage of proteins with MW greater than 60 kDa in the spores 4 hag. The percentage of spots with MW less than 30 kDa increased by ~6% from 0 to 2 hag. and decreased by 10% from 2 to 4 hag. (Additional file 2: Figure S1). The images show predominance of acidic proteins (isoelectric points less than 5.5) in nongerminated basidiospores compared to germinated spores. On the other hand, the number of basic proteins (isoelectric points greater than 7.5) in nongerminated spores was generally less than the number of basic proteins detected in germinated spores. Although the gels for 2-h and 4-h samples do not have spots in all pH ranges seen on the 0-h gels, the distribution of proteins on the basis of their pI was homogeneous in the ranges that contained spots (Additional file 3: Figure S2).

**Fig. 2:**
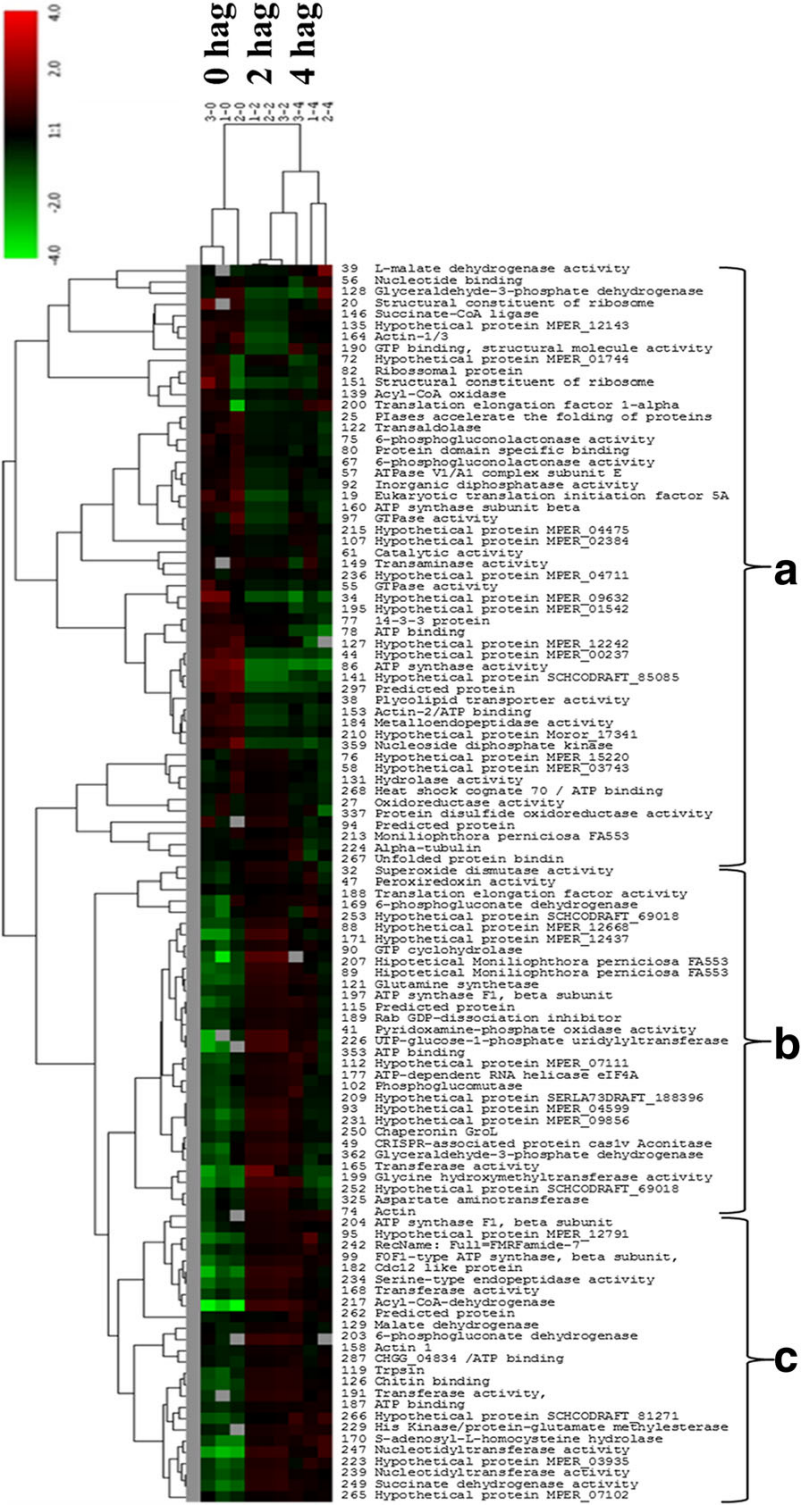
Bi-directional Hierarchical Clustering Analysis generated by Cluster 3.0 software showing the global profile of differential expression of proteins common to the three germination times. **a** Proteins repressed 4 h after germination. **b** proteins repressed 2 h after germination. **c** Proteins induced 4 h after germination

A total of 109 spots common for all three treatment groups, as identified by MS/MS, were analyzed and clustered according to their profile. Proteins that showed similar accumulation patterns were grouped by hierarchical clustering (Fig. 2). Group A contains proteins that showed reduced expression with time after germination. Group B contains proteins that showed an increase in expression 2 hag. but then were downregulated at 4 hag. Group C contains proteins that showed only accumulation during germination. More information on the identification of spots can be found in Additional file 1: Table S1.

### Most spots with high molecular weight correspond to enzymes associated with metabolic processes

**Fig. 3:**
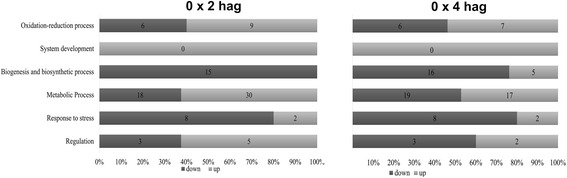
Classification of biological processes for differentially expressed proteins identified in non-germinated spores of *M. perniciosa*, compared with 2 and 4 h after germination. Classification was performed using Blast2GO software

Unique and differentially expressed proteins were classified into categories by their biological processes as determined by the Blast2GO software. Some categories were chosen to be discussed because of their differential accumulation between 2 and 4 hag, relative to nongerminated spores as a reference [20]. The categories chosen were oxidation-reduction, development, biosynthetic, and metabolic processes, the stress response, and regulation. When compared with nongerminated spores, 40% (6) and 46.1% (6) of proteins related to oxidation-reduction were downregulated in treatment groups 2 and 4 hag., respectively. Two proteins related to fungal development showed accumulation at 2 hag., and one was upregulated 4 hag. Among all the proteins detected in nongerminated spores, 15 were classified into the category related to biogenesis and biosynthetic processes yet none of these proteins appeared at 2 hag. Four hag., 79.2% of biosynthetic proteins showed reduced accumulation, and 20.8% showed increased accumulation when compared with nongerminated spores. The category related to metabolic processes was the most represented, with 40% of the proteins showing reduced accumulation and 60% showing increased accumulation 2 hag., and at 4 hag., 52.7% of proteins showed reduced accumulation and 51.3% showed increased accumulation. Among the proteins involved in stress response, 80% of proteins showed reduced accumulation and 20% showed hyperaccumulation at 2 and 4 hag., respectively. At 2 hag., 62.5% of the proteins involved in regulation showed increased accumulation, and at 4 hag., 60% of the proteins showed decreased accumulation (Fig. 3).

The increase in the amount of high-molecular-weight proteins in spores 4 hag may be related to the increased expression of energy production machinery. Most high-molecular-weight spots correspond to enzymes associated with metabolic processes, for example, ATP synthase (68.1 kDa), ATP-binding protein (68.8 kDa), and succinate dehydrogenase (62.3 kDa).

The distribution of spots in gels is a qualitative result that facilitates the choice of pH and MW range in the subsequent studies. Twenty acidic proteins were identified by mass spectrometry. Acidic-protein enrichment techniques have been tested on the samples in 2D electrophoresis to analyze differences in the accumulation between acidic subproteomes of yeast and hyphae in *Candida albicans* [27]. This analysis can also be conducted to identify proteins in acidic subproteomes of phytopathogenic fungi such as *M. perniciosa* and *M. roreri*. The acidic proteins identified in the present study participate in regulation of important molecular functions (spots 15, 36, 77, 80, and 88), cytoskeleton organization (spots 97 and 305), energy metabolism (spots 19, 160, 184, 195, 304, and 700), and a stress response (spots 270, 337, 714, 751, and 811). Among these proteins, the only ones related to energy metabolism that were downregulated 2 and 4 hag. were translation initial factor C (spot 19) and ATP synthase β subunit (spot 160), respectively.

### Proteins related to metabolism and energy

Proteins associated with protein metabolism showed progressively decreasing abundance throughout germination: translation initial factor (spot 19) and nucleoside diphosphate kinase (spot 297) decreased at 2 hag. (Additional file 5: Table S2), and small ribosomal subunit (spot 109) and heat shock protein (spot 263) decreased 4 hag. (Additional file 5: Table S2). Two other proteins showed reduced expression after the onset of germination (spots 34 and 44; Fig. 2a). Although these proteins were not characterized in the *M. perniciosa* database, BLASTP analysis showed that these spots correspond to proteins participating in the metabolism of proteins, e.g., 60S ribosomal protein (spot 34) and serine protease inhibitor (spot 44). This finding may be related to the stress response and synthesis of new proteins when a basidiospore prepares for the growth of the germinative tube [20]. In addition, several heat shock proteins and other stress-related proteins have been demonstrated to improve the ability of nongerminated conidia to survive various types of environmental stress [28].

**Fig. 4:**
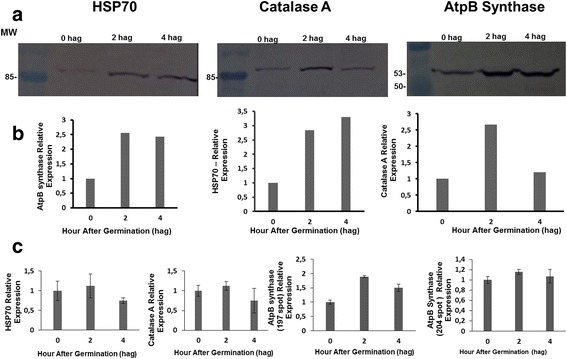
Accumulation of BiP, Catalase, and ATP synthase (beta chain) by western blot and analysis of spots. **a** Image of a nitrocellulose membrane hybridized with Anti-BiP, Anti-Catalase, and Anti-ATP synthase (beta chain) of Arabidopsis (Normalization, see Additional file 6: Figure S4). **b** Relative accumulation determined from images of the nitrocellulose membrane, using the Gel Quant. Net 1.8.0 software. **c** Relative quantification of each spot corresponding to proteins analyzed by western blot. The average values of the each spot’s percentage volume in each gel replicate was used for graph construction

Other proteins related to protein metabolism were induced, and some were exclusively detected at 4 hag. (Fig. 3). These proteins include nucleoside triphosphate hydrolase proteins (spot 384), constituents of larger subunits of ribosomes (spot 397), translation elongation factor 1α (spot 383), folding protein (spot 390), and HSP70 (spot 522; Additional file 1: Table S1). Compared to nongerminated basidiospores, BiP showed upregulation of ~2.8-fold at 2 hag. and ~3.4-fold at 4 hag. BiP upregulation correspond to the expression of a few spots related to HSP70 (spots 268 and 278) that were common among the treatments. As expected, the final germination phase of *M. perniciosa* basidiospores requires expression of some of these proteins for transcription and synthesis of new proteins. In addition, upregulation of BiP (a molecular chaperone from the HSP70 family), i.e., Spot 278, resident in the endoplasmic reticulum (ER), occurred 4 hag. as shown by the cross-reaction with the antibody against BiP of *A. thaliana* (Fig. 4a and b). BiP also plays a central role as a sensor of environmental stressors in the ER, affecting the assembly and folding of proteins [29, 30]. BiP induction during the germination of *M. perniciosa* is suggestive of an increase in ER secretory activity [31]. This finding is in agreement with proteomic studies on *A. nidulans* germination, where multiple heat shock proteins were found to be upregulated 30 and 60 min after the onset of germination [14].

Proteins with increased expression during germination were clustered (Fig. 2b). Among these, six are members of the ATP synthase family (spots 204, 95, 242, 99, 187, and 266). Accumulation of ATP synthase can be noted in western blot analysis (spots 197 and 204) performed in this study. These proteins may be related to a direct increase in metabolic activity and energy production needed to meet the energy demand for the development of the germinative tube. Conversely, glyceraldehyde-3-phosphate dehydrogenase (spot 128, Fig. 2a) was downregulated after the onset of germination. This phenomenon may be related to the temporary accumulation of glycerol during germination. The same phenomenon has been observed in *Phycomyces blakesleeanus* and seems to be caused by the activation of cAMP-dependent glycerol-3-phosphatase: an enzyme that indirectly participates in the glycolytic pathway [32] Excessive increase of glycerol production during spore germination can cause a buildup intracellular osmotic pressure [33]. This observation may explain the swelling observed in *M. perniciosa* basidiospores before the start of the germinative tube emission, i.e., from 0 to 30 min after defrosting and introduction of basidiospores [20].

### Cell cycle-related proteins and septation of the primary hypha

Valosin-containing protein (VCP) was identified (spot 304), which is a member of the AAA ATPase family (ATPases associated with a variety of activities) and contains two conserved domains also called AAA [34]. VCP is a homolog of CDC48p, which is well conserved among all eukaryotes and is essential for growth of *Saccharomyces cerevisiae* [7]. This protein performs a critical function in regulation of the cell cycle, and is required for entry into mitosis [35]. During the germination of basidiospores, this protein can be associated with the process of cell division and consequently with the growth of hyphae. Similarly, a kinesin (spot 395) was identified, which is a motor protein with an important role in cell division. Kinesins are necessary for proper length and sliding of microtubules inside the spindle during prophase and metaphase and for microtubule depolymerization during anaphase [36].

Septin (spot 488) is another protein found only in spores 4 hag. Since its discovery in *S. cerevisiae* [37], other septins have been found in eukaryotes, especially in filamentous fungi, where septins control the filamentous morphology. During septum formation, the septin ring splits in two to form a double ring. Due to their lack of septa, septin mutants are highly sensitive, and damage to a single hypha can result in complete lysis of a young mycelium [38]. In studies conducted on phytopathogenic basidiomycete *U. maydis*, gene *Sep3* was found to perform important functions in morphogenesis of the filamentous cells, in the corn infection process, and in symptom development [39]. In *Magnaporthe oryzae*, septins mediate cortex rigidity and curvature of the membrane necessary for spore penetration of a rigid plant part, like the cuticle of a leaf, to facilitate infection [40]. To analyze septin function, some targeted drugs can be used; however, for the most part, these compounds are not readily available. One such compound is forchlorfenuron (FCF; N-[2-Chloro-4-pyridyl]-N′-phenylurea; HPM300), a plant cytokinin able to quickly induce production of abnormal septin structures in *S. cerevisiae* [41]. To gain an insight into how FCF binds to septin proteins, simulations were carried out in silico on the basis of docking of FCF’s structure to all the high-resolution crystal structures of septin available. These studies suggested that FCF mimics nucleotides and thus interferes with the dynamics of GTP-binding proteins by modifying the mounting base of septin filaments [42]. In this regard, we recommend that the septin protein be used in studies on effective control of the invasion of young tissues of cacao plants by fungus *M. perniciosa*.

### The response to stress-related proteins

Proteins active in the stress response are present among the ones detected exclusively in nongerminated basidiospores. Oxidoreductases (spots 684 and 687) and ascorbate peroxidase (spot 688) belong to this category (Fig. 3). These proteins may be involved in a fungal adaptation intended to neutralize reactive oxygen species (ROS) produced by plants as a defense mechanism [23]. In addition, other studies have indicated that oxidative respiration is absolutely required for germination of fungal spores [43, 44].

The relative expression of catalase A detected in 2D gels of proteins at 0, 2, and 4 hag. correlates with the expression of this protein detected by western blotting with an anti-CatA antibody specific for the *A. thaliana* protein (Fig. 4a–c). An increase in catalase expression can be observed 2 hag. This change may contribute to the survival of basidiospores and mycelium of the biotrophic type of *M. perniciosa* treated with 4 mM hydrogen peroxide in vitro [45]. In addition, there was upregulation of SOD (spot 32) at 2 hag. (Table 1). SOD expression, combined with the accumulation of peroxiredoxin (spot 47; Fig. 2b) and catalase, points to the need for neutralization of free radicals within the germinative tube during germination. Similarly, accumulation of catalase A during the germinative process of *A. nidulans* in the first 30 min after germination has been observed elsewhere [14].

### Fungal-virulence-related proteins

Peptidyl-prolyl cis-trans isomerase (spot 695) is a relevant protein that was detected exclusively in nongerminated spores (Additional file 1: Table S1). This protein is associated with the pathogenicity of *B. cinerea*, being characterized as a virulence factor important for host invasion [46]. These data suggest that nongerminated basidiospores of *M. perniciosa* accumulate virulence factors essential for a quick attack on the host in a manner similar to the mode of action of *B. cinerea* conidia [23].

Our results suggest that a secondary terpenoid metabolite synthesis route associated with polyketides can be enabled in *M. perniciosa* during germination [47]. Transcription factor FapR (Table 1, spot 323), which regulates both clusters of genes involved in the biosynthesis of secondary metabolites fumagillin and pseurotin in *Aspergillus fumigatus* [47], was induced 2 hag. The metabolite fumagillin is a hybrid polyketide-terpenoid compound [48]. Additionally, polyketide synthase (gi|392,596,225) was identified exclusively 4 hag. (Table 2, spot 388). Polyketide synthase belongs to a large family of enzymes responsible for production of various secondary metabolites such as pigments, toxins, antibiotics, and signaling molecules [49]. This protein catalyzes the first reaction of melanin biosynthesis [50] and may participate in the synthesis of fumagillin [47]. Melanin production by pathogenic fungi can contribute to their virulence toward humans and cultivated plants [51].

## Conclusion

**Fig. 5:**
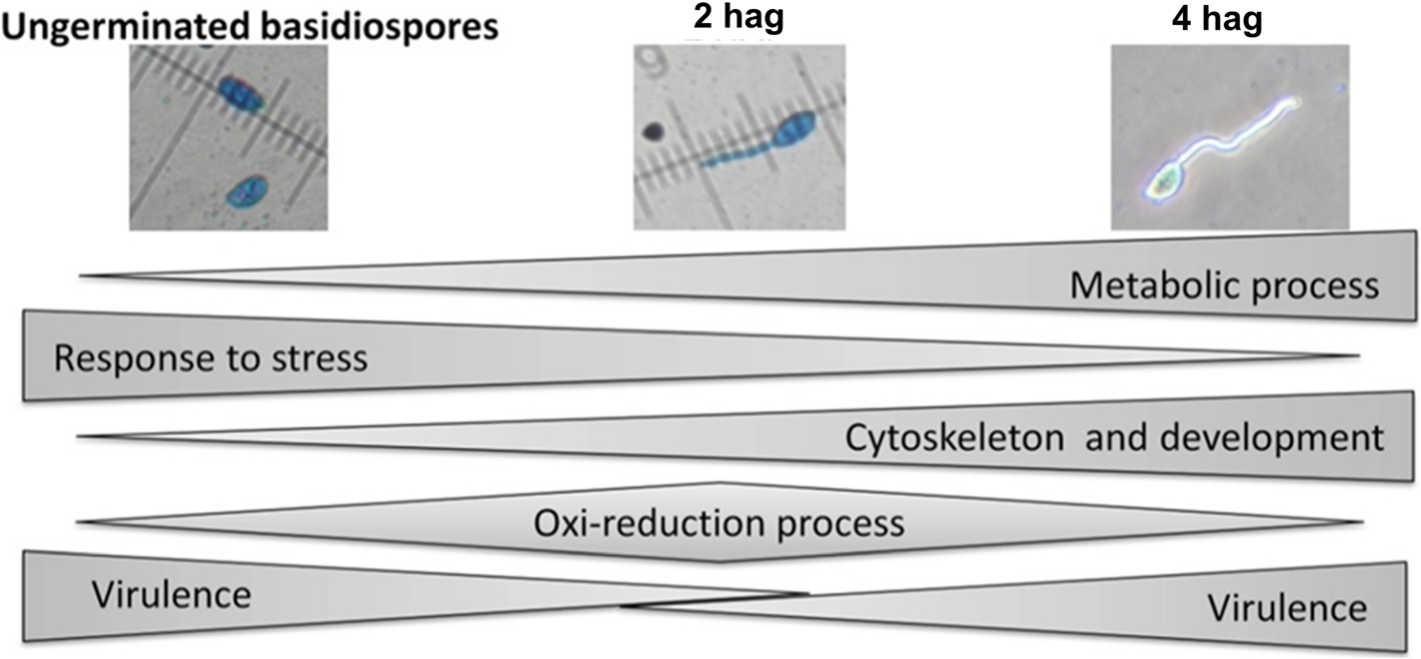
General scheme of the distribution of proteins according to their biological process, over the first 4 h of *M. perniciosa* basidiospores germination

Generally, the comparative proteomic analysis based on a combination of 2D–SDS-PAGE with MS/MS enabled identification of a general scheme of biological processes that take place during the initial developmental phase of fungus *M. perniciosa* (Fig. 5).

In conclusion, in this study, we showed variations in protein expression at the early germination stages of *M. perniciosa*. Some proteins related to metabolism are expressed as the germinative tube develops. Protein synthesis intensifies during germination, judging by the accumulation of ribosomal components, elongation factors, and BiP. Upregulation of oxidative-stress-related proteins such as SOD and catalase can facilitate detoxification of free radicals inside the primary hypha. Proteins associated with fungal filamentation, and consequently with virulence, were detected in basidiospores 4 hag., for instance, septin and kinesin. This finding indicates that the germinative tube is preparing for primary hypha differentiation and host invasion. These proteins are promising targets for strategies of fungal control, when inhibitory compounds become available. Additionally, it is possible that pathogenicity-related proteins are specific to each step of the process of fungal infection. Virulence factors such as polyketide synthase and FapR are present in basidiospores and the primary hypha. Accordingly, we propose a model of the germination of fungus *M. perniciosa* (Fig. 5). This research may help to unravel the mechanisms behind the basic processes that this pathogen evolved to survive in (and to invade) the host tissue, causing one of the diseases responsible for huge losses in cacao cultivation in South and Central America.

## Additional files

Additional file 1: Table S1. Ratio values for each protein spot used to perform the hierarchical clustering analysis. (XLS 61 kb)

Additional file 2: Figure S1. Sequence alignment of the target proteins identified in the western blots and their homologous in plant recognized by the antibodies. A – Sequence alignment between BiP from *A. thaliana* and the HSP70 from *Moniliophthora perniciosa*. B – Sequence alignment of the ATP synthase from *A. thaliana* and its homologous in *Moniliophthora perniciosa*. C – Sequence alignment of the catalase A from *A. thaliana* and its homologous in *Moniliophthora perniciosa.* (JPEG 3987 kb)

Additional file 3: Figure S2. Distribution of the spots and their respective molecular weight in the 0, 2 and 4 hag. (TIFF 2477 kb)

Additional file 4: Figure S3. Distribution of the spots and their respective isoelectric point in the 0, 2 and 4 hag. (TIFF 741 kb)

Additional file 5: Table S2. Total spots identified in the MS/MS. In sequence, mw corresponds to a molecular mass values, the isoelectric point pI estimated; number of peptides by MS / MS; Score of sequence, sequence coverage and peptide sequence. All values were calculated by Mascot at http:. //www.matrixscience.com. (XLS 118 kb)

Additional file 6: Figure S4. Background detection by western blotting by overincubating the membranes. The anbidodies against Catalase and HSP70 were very specific. However, the antibody against Catalase has revealed background bands that were homogeneously transferred to the membrane at 0, 2 and 4 hag. (TIFF 498 kb)

## Abbreviations

2D: Two-dimensional
BiP: Binding immunoglobulin protein
hag: Hours after germination
LC-MS/MS: Liquid chromatography with tandem mass spectrometry
PAGE: Polyacrylamide gel electrophoresis
SOD: Superoxide dismutase
