# Supplementary figures and images for: Proteomic analysis during of spore germination of Moniliophthora perniciosa, the causal agent of witches’ broom disease in cacao
Source: BMC Microbiol. 2017 Aug 17;17:176. doi: 10.1186/s12866-017-1085-4 (PMC5561645; doi:10.1186/s12866-017-1085-4)

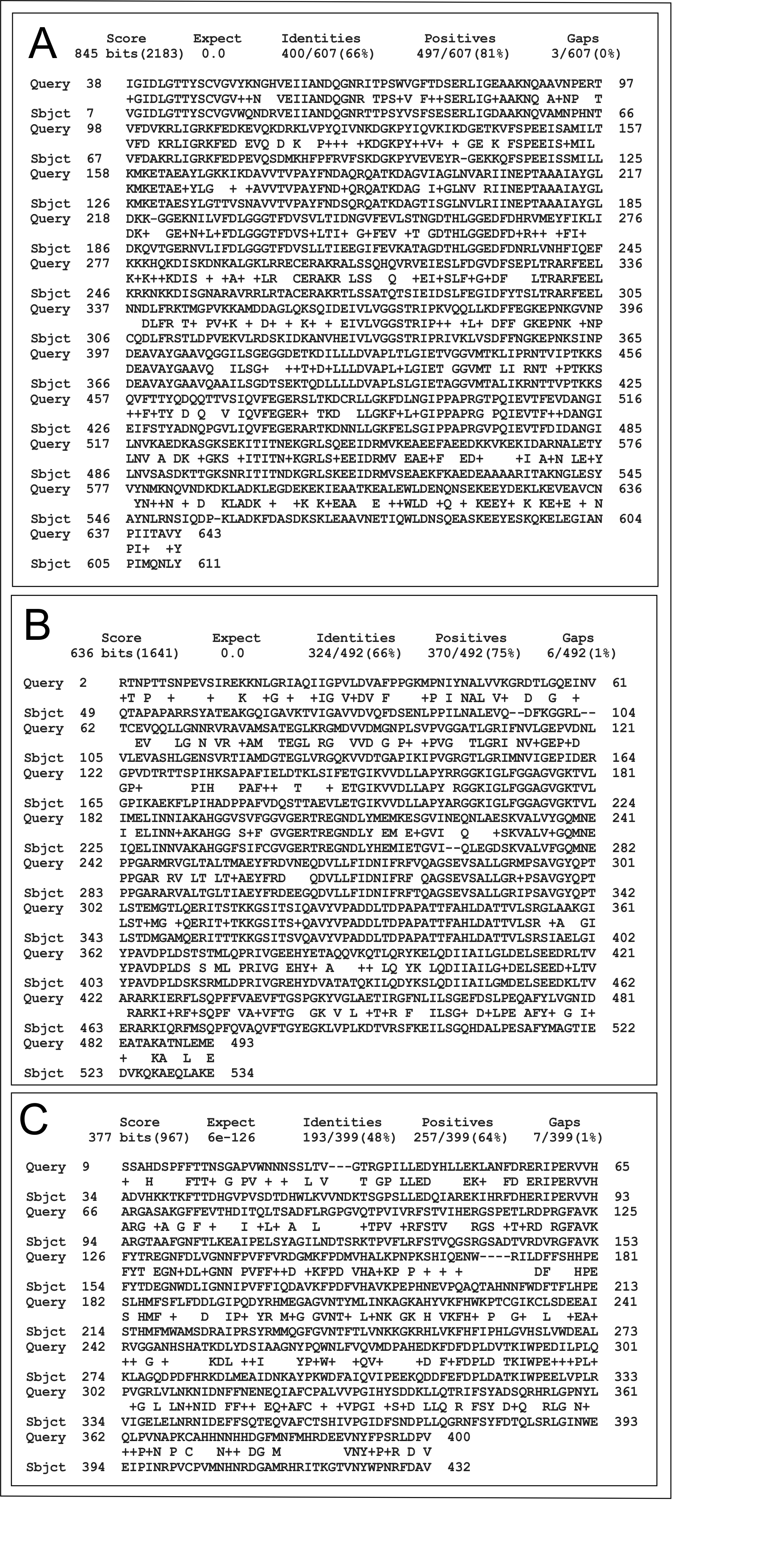

Supplement: Supplementary file 2 — Sequence alignment of the target proteins identified in the western blots and their homologous in plant recognized by the antibodies. A – Sequence alignment between BiP from A. thaliana and the HSP70 from Moniliophthora perniciosa. B – Sequence alignment of the ATP synthase from A. thaliana and its homologous in Moniliophthora perniciosa. C – Sequence alignment of the catalase A from A. thaliana and its homologous in Moniliophthora perniciosa. (JPEG 3987 kb) [file 12866_2017_1085_MOESM2_ESM.jpg]

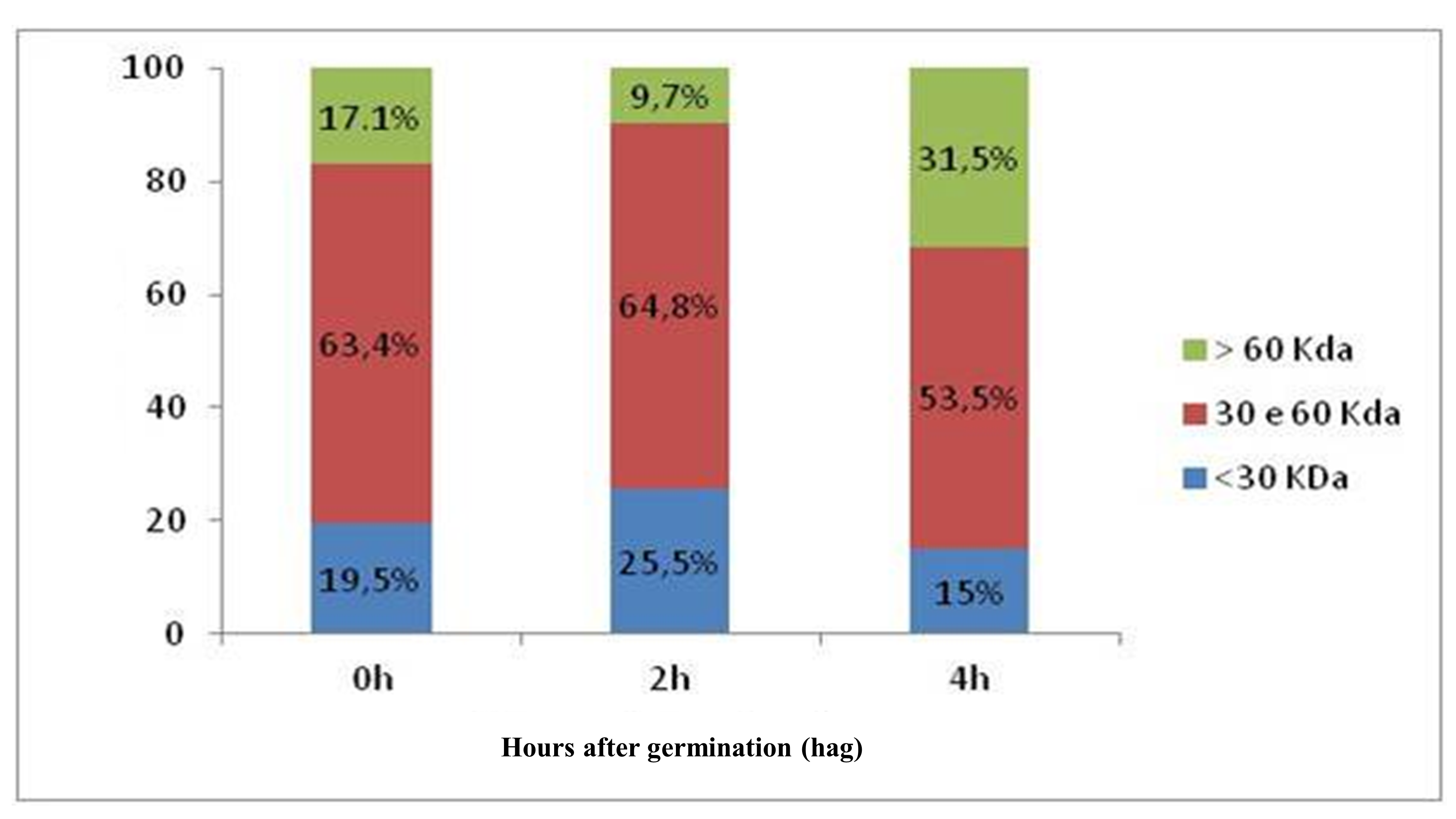

Supplement: Supplementary file 3 — Distribution of the spots and their respective molecular weight in the 0, 2 and 4 hag. (TIFF 2477 kb) [file 12866_2017_1085_MOESM3_ESM.tif]

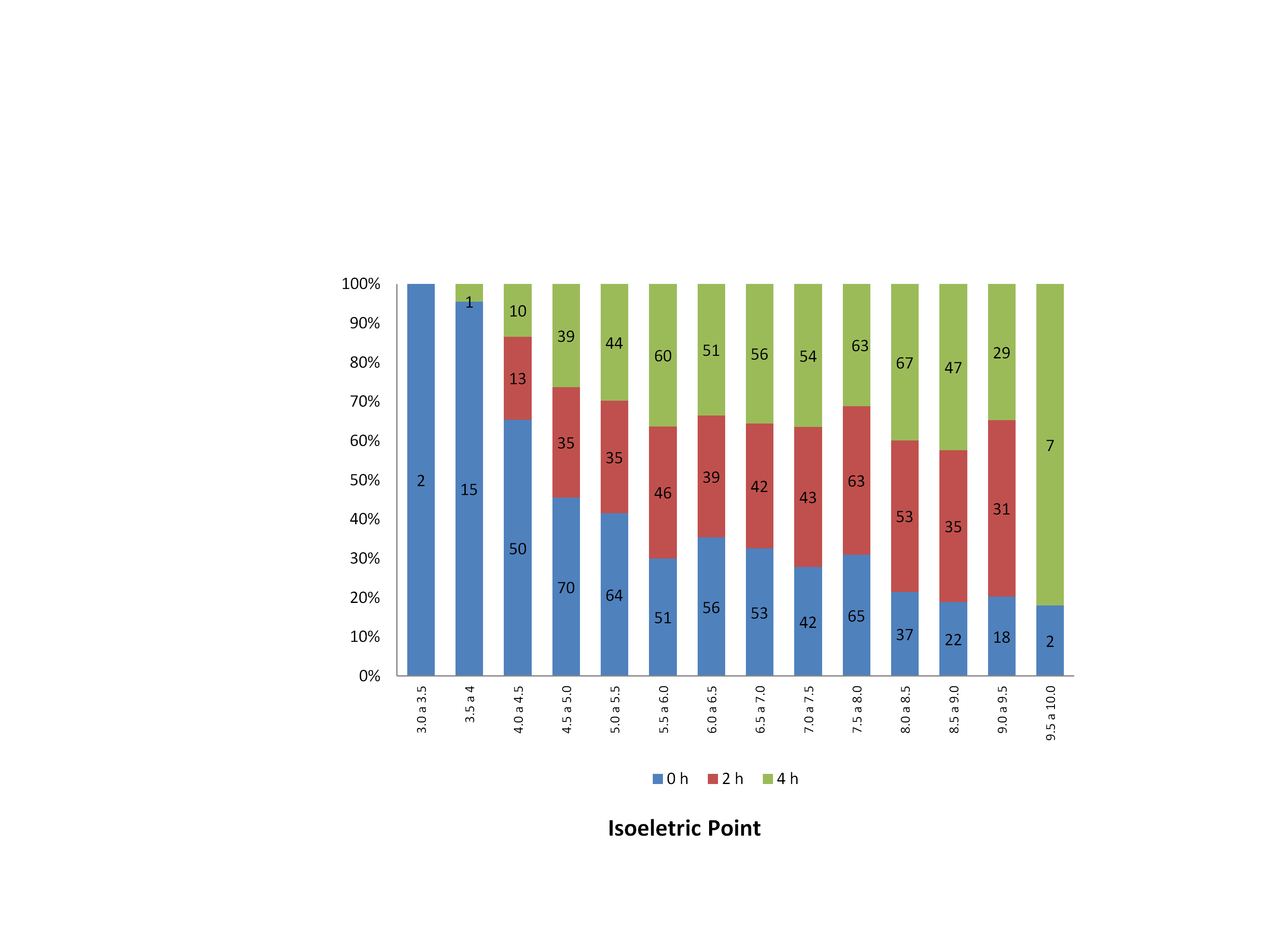

Supplement: Supplementary file 4 — Distribution of the spots and their respective isoelectric point in the 0, 2 and 4 hag. (TIFF 741 kb) [file 12866_2017_1085_MOESM4_ESM.tif]

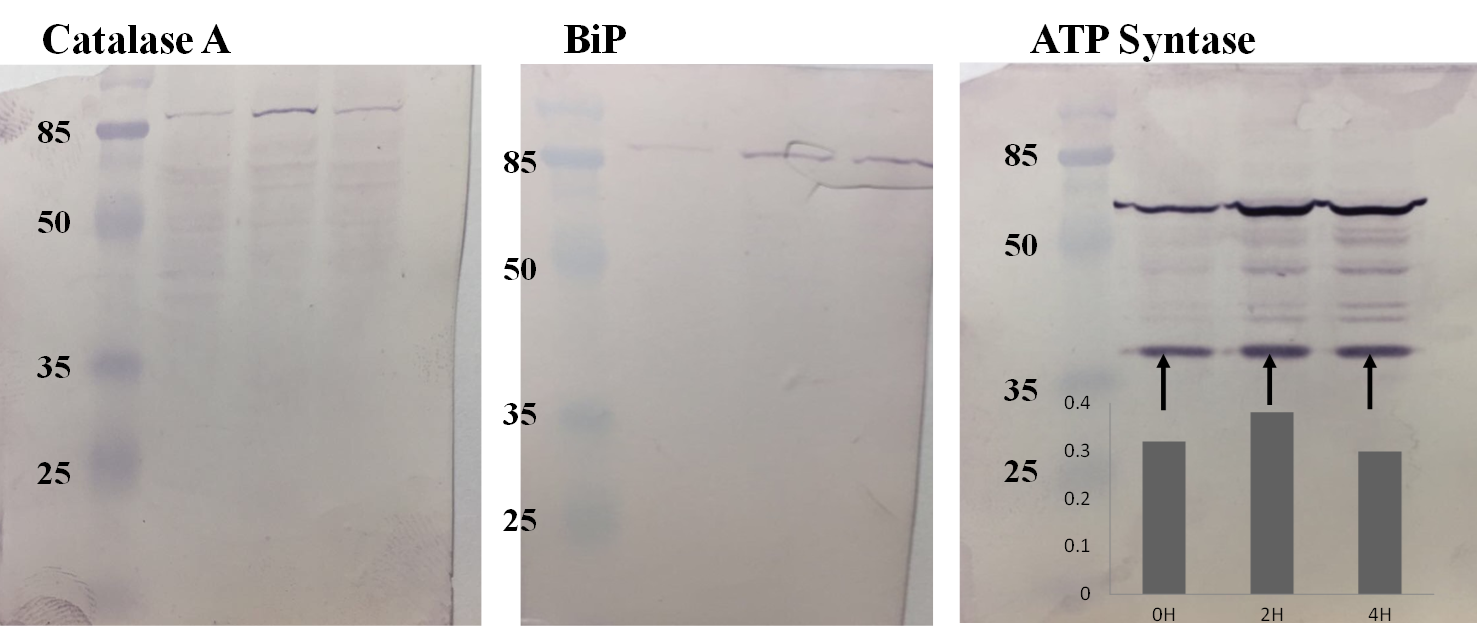

Supplement: Supplementary file 6 — Background detection by western blotting by overincubating the membranes. The anbidodies against Catalase and HSP70 were very specific. However, the antibody against Catalase has revealed background bands that were homogeneously transferred to the membrane at 0, 2 and 4 hag. (TIFF 498 kb) [file 12866_2017_1085_MOESM6_ESM.tif]
